# The appropriateness of psychotropic medicines: an interview study of service users attending a substance misuse service in England

**DOI:** 10.1007/s11096-019-00861-z

**Published:** 2019-06-13

**Authors:** Adejoke Obirenjeyi Oluyase, Duncan Raistrick, Elizabeth Hughes, Charlie Lloyd

**Affiliations:** 1 Department of Health Sciences, University of York, Area 4 ARRC Building, Heslington, York, YO10 5DD UK; 2 Cicely Saunders Institute of Palliative Care, Policy and Rehabilitation, King’s College London, London, SE5 9PJ UK; 3 Leeds and York Partnership NHS Foundation Trust, 19 Springfield Mount, Leeds, LS2 9NG UK; 4 School of Healthcare, Faculty of Medicine and Health, University of Leeds, Leeds, UK

**Keywords:** Addiction service, Appropriateness, Mental Health, Psychotropic medications, Specialist addiction service, United Kingdom

## Abstract

*Background* Mental health problems are common in people with substance misuse problems. However, there is a paucity of evidence regarding prescribing of psychotropic medications for people with comorbid mental health and substance misuse problems. *Objective* To explore the views of service users attending an addiction service on the appropriateness of psychotropic medications prescribed for their co-existing mental health problems. *Setting* A specialist addiction service in the North of England. *Method* A phenomenological approach was adopted. Semi-structured interviews were conducted with twelve service users. Data were analysed using thematic framework analysis. *Main outcome measure* Service users’ views concerning the appropriateness of their prescribed psychotropic medications. *Results* The following themes captured service users’ views on the appropriateness of their medications: benefits from medicines, entitlement to medicines, and assessment and review. Service users mostly described benefits from their medications (including those prescribed outside guideline recommendations) and there was also an awareness of the adverse effects they experienced from them. It appears that people with substance misuse problems have a particularly strong sense of their own needs and seek to influence prescribing decisions. Service users further described varied practices regarding assessment and review of their medications with evidence of regular reviews while others identified suboptimal or inadequate practices. *Conclusion* Most service users described improved functioning as a result of their prescribed psychotropic medications. Prescriptions that are inappropriate in terms of their usual indications may well be justified if they assist in stabilising service users and moving them on to recovery.

## Impacts on practice

- Addiction service users appear to have a strong attachment to their psychotropic medications and a belief that they have a right to be prescribed whatever they feel they need.
- Prescriptions that are inappropriate in terms of their usual indications for users of specialist addiction services may be justified if they provide benefits and can be used to move people on to recovery.

## Introduction

Lexchin [1] has described appropriate prescribing as ‘trying to maximise effectiveness, minimise risks and costs, and respecting service users’ choices [1]. Of importance in judging prescribing appropriateness are what the service users wants or prefers as well as the scientific rationalisation [2, 3].

Among people with substance misuse problems, their wider physical, psychological and social outcomes as well as their views may be particularly important when assessing the appropriateness of their medications. People with substance misuse problems often have complex circumstances encompassing health, social and economic problems [4, 5], with service users attending specialist addiction clinics being more likely to have higher levels of dependence, multi-morbidities, functional impairment, social problems and use of multiple medications when compared with those who are not help-seeking [6, 7]. These complex needs may influence prescribing decisions in this population [7, 8]. For example, people with co-existing substance misuse problems and mental disorders may need higher doses or longer duration of treatment because they may have more severe and persistent symptoms or they may be more resistant to treatment [9, 10].

Most of the published research on prescribing appropriateness has focused on the assessment of pharmacological appropriateness [3]. Examples of measures used for assessing this include the medication appropriateness index (MAI) [11] and the prescribing appropriateness index (PAI) [12]. Moving beyond the medical perspective by considering service users’ preferences and context, introduces subjectivity into decision-making concerning appropriateness. Barber et al. [3] have argued that the introduction of subjectivity is appropriate when making value judgments such as this.

Furthermore, given that intoxication and withdrawal symptoms of substances often mimic those of anxiety and mood disorders, an important clinical question relates to whether and when psychotropic medications could be used in treating the mental health symptoms of people with substance misuse problems [13–15]. There is inconsistent evidence that psychotropic medications such as antidepressants and sedative/hypnotic drugs have beneficial effects on co-existing mental disorders in this population [16–19].

It has been suggested that the service user’s perspective is particularly important and should be considered where there is a lack of adequate evidence or doubt about the best course of action [20, 21]. In the UK, the NHS constitution states that the service it provides must reflect the needs and preferences of service users [22]. Partnership with service users with open communication and consideration of their values and choices have been promoted in UK government policies and clinical guidance [23, 24], with positive effects reported on satisfaction, medication adherence and well-being. Additional benefits described among those with substance misuse problems include increased personal control and reduced drug use [25, 26]. Generally, service users want to be involved in decision-making concerning their treatment and medications [26]. However, they often report feeling uninvolved in decision-making [27]. A qualitative study that explored the experiences of service users in treatment for alcohol use disorders around their treatment needs and satisfaction while also comparing the experiences of those with and without co-morbid severe mental health symptoms (SMHS) found that those with comorbidity described a need for medications to relieve their psychological symptoms and were often dissatisfied when they were left out in the decision making process (including prescribing) [27]. Although this study described the treatment needs of service users with comorbid SMHS, it was not focused on the appropriateness of prescribed psychotropic medications. No previous study has explored the views of people attending an addiction service concerning the appropriateness of psychotropic medications prescribed for their co-occurring mental illness. Consequently, this study was carried out to address this gap. Diagnoses of mental health illnesses and psychotropic medications were self-reported by service users.

This study was part of the first author’s (A.O) PhD programme. As a pharmacist, A.O has always had an interest in the quality of prescribing for service users, including those with substance misuse problems. A.O’s pharmacy training with emphasis on the use of medications for the management of substance misuse problems made her initially more inclined to view these disorders as brain diseases. Hence, A.O came with a medical perspective of substance misuse problems when she started her doctorate programme.

### Aim

To explore the perspectives of service users in a specialist addiction service on the appropriateness of psychotropic medications prescribed for their co-existing mental health problems. Appropriateness was explored by assessing service users’ views concerning the following areas: their need for medications, medication effectiveness, medication changes, assessment and review.

### Ethics approval

The study was approved by the University of York’s Research Governance Committee and the National Research Ethics Service (NRES) Committee Yorkshire & The Humber. Reference 12/YH/0325.

## Methods

### Study design and setting

Service users’ views were explored using a phenomenological approach. Phenomenology is based on the assumption that reality is multiple and socially constructed through the interaction of individuals with others and the world around them [28]. Semi-structured qualitative interviews were carried out with 14 service users who were attending the specialist addiction service. The specialist addiction service has been described in a previous study [7]. Briefly, it is located in a city in the North of England and is a statutory NHS specialist service that provides a full range of psychosocial and pharmacotherapies for addiction problems. All interviews were carried out at service users’ three-month follow-up appointment or at their next appointment if this was more convenient. Interviews were carried out with a single service user at a time due to the sensitivity of the topic and to maintain confidentiality [29].

### Participants

Interviews were carried out with a convenience sample of 14 service users on current prescriptions of psychotropic medications. Of these 14, two service users provided very limited information and their data were not presented. Only data from 12 service users were reported. Psychotropic medications included in this study were antidepressants, anxiolytics/hypnotics, anticonvulsants, antipsychotics and mood stabilisers. All service users who were interviewed also took part in an earlier study [30] in which the appropriateness of opioids and psychotropic medications were assessed using a modified form of the Medication Appropriateness Index [11]. Convenience sampling involves recruiting people who are available to take part in research [31], and may not be representative of the study population [32]. Service users who were still attending the specialist addiction service prior to their three-month follow-up appointment and who were on prescribed opioids and/or psychotropic medications at their first assessment were contacted by telephone to determine if they could be interviewed during their follow-up appointment. Twenty-three service users were on these prescribed medications and 20 of them were contacted by telephone to determine if they would consent to participate in an interview during their follow-up assessment. The remaining three service users could not be contacted. Four service users were not interviewed because they did not attend for their scheduled interviews while two service users attended but declined interview. Therefore, a total of 14 service users (of 20:70%) were interviewed and data from 12 of them are presented (60%).

Service users who provided consent and attended the service for their follow-up appointment were reminded of the study aims and encouraged to ask for clarification where needed. They were then given a consent form to sign before the interviews commenced. The service users who were interviewed comprised two females and 10 males. Alcohol was the referral substance for all but two service users who were referred for problems with heroin. The mean age of those interviewed was 48.6 years (range: 26 to 66 years) and the number of psychotropic medications they were being prescribed at their three-month assessment ranged from 1 to 4.

### Data collection

Data were collected by the first author, A.O. All the interviews were conducted in a private room at the specialist addiction service between October 2012 and April 2013 and lasted on average 47 min (range: 15 to 104 min). The topic guide for service users was developed from the research questions and advice from the project advisory group. It covered the following areas: service users’ medical/substance use history, need for medications, effectiveness of medications, assessment and review, medication change and quality of life. The views of ex-service users who serve as mentors for other service users at the specialist addiction service were sought about the topic guide. All interviews were audio-recorded and transcribed verbatim.

### Data analysis

Thematic framework approach was used for data analysis [33]. Repeated reading of the transcripts alongside listening to the audio-recordings ensured that the authors were familiar with the data. This was followed by a period of descriptive and interpretive coding facilitated by Atlas ti (v 6.0). This inductive approach enabled a deeper understanding of the data [33]. The coding framework was expanded as new themes emerged. Broader themes were subsequently generated and frequently reviewed while comparing data from participants that supported the themes and also looking for explanations of any differences of viewpoints within the data. In order to ensure anonymity and confidentiality, numbers rather than names were allocated to participants. Trustworthiness of the data was ensured through an audit trail kept by A.O which detailed how data were collected, how themes were formed and how decisions were made during the research process. Given A.O’s initial views on substance misuse problems as brain diseases, the interpretation of the data was discussed in-depth with two of the authors (C.L and E.H), who reflected on the emerging themes and the depth of the analysis. C. L and E.H studied criminology and nursing respectively. D. R is a consultant addictions psychiatrist.

## Results

Three main themes emerged by undertaking a thematic analysis: benefits of medicines, entitlement to medicines, and assessment and review. Two subthemes, medication review and discussion with clinicians, are further described under assessment and review.

### Benefits of medicines

This theme emerged in response to service users’ views concerning the need for their medicines and effectiveness. All service users described their medicines in relation to the effect they had on their health conditions and wider life. Most service users thought that their medicines had led to some degree of improvement in their health conditions and functioning. Below is a description of the positive impact of antidepressants on a service user’s functioning:

…because a few months ago, I didn’t take them [Citalopram] for a while and I just hit rock bottom again and then when I started back on them again, within a couple of weeks I was back to normal [ID 5, male, heroin dependency].

As well as the health impact of their medicines, service users also described their medicines (in particular, antidepressants) as providing valuable support in their lives:

…and during a very stressful period which, my mood didn’t really change that much, I used it as like a comfort blanket or a crutch to sort of like, I’m taking 40 mg [Citalopram] now, so I’m bound to be all right [ID 23, male, alcohol dependency].

Among service users who described positive effects from their medicines were those who reported benefits despite being prescribed outside the British National Formulary (BNF) recommendations. One elderly service user referred for his alcohol problems, with a 12-year history of olanzapine use at a dose above the BNF recommendation for long-standing paranoid schizophrenia, described the benefit he derived from his medicine:

As soon as I was put on 25 mg of Olanzapine a day, at night, all slight psychotic symptoms disappeared. And I’ve been on those tablets for a number of years now and I find that they do very well for me psychiatrically. I haven’t been mentally ill for something like oh about ten/twelve years now… thanks to the medications [ID18, male, alcohol dependency].

There was also an awareness that alcohol could have a negative impact on the effectiveness of their antidepressants. The service user with long-standing paranoid schizophrenia described above recounted periods of depression despite being on fluoxetine and mirtazapine. He attributed this to continued drinking.

Another service user with a year history of zopiclone use for sleep problems described benefit from his zopiclone (the duration of zopiclone use had exceeded BNF recommendations):

The Zopiclone works because within ten, twenty minutes I’m asleep. So that does work [ID 15, male, alcohol dependency].

Service users further described having side effects which they tolerated due to the benefits they felt they obtained. One service user with a five-year history of citalopram use for long-standing depression described sexual dysfunction resulting from citalopram but reported that it was nevertheless effective for his depression:

Citalopram’s been great. Easy to work with, you know, easy to take, it doesn’t make me drowsy. I can live a normal life on it, and I’m happy now. The only contraindication or side-effect I have from citalopram is that sometimes it prevents ejaculation. I can get to the point of climax but, I don’t ejaculate [ID 23, male, alcohol dependency].

Almost all service users who had changes made to their medicines described improved functioning as a result of this.

### Entitlement to medicines

While describing the need for their medicines and its effectiveness, service users went ahead to describe a sense of entitlement to being prescribed medicines:

Well my personal opinion is that I think with doctors these days, I think you have to tell them what’s wrong with you or basically tell ‘em what you need, rather than the sort of old school where you went to the doctor and you spent a long time explaining your symptoms (ID 7, male, alcohol dependency).

I do appreciate that they [Zopiclone] are addictive but so’s smoking, so’s driving really fast, so’s doing a lot of other things. It’s my health and I’ve chosen to look after it how I want and this is how I want (ID 57, male, alcohol dependency).

This theme was also evident when another service user who had deliberately taken an overdose of his prescribed antidepressant (fluoxetine) and sleeping tablet (zopiclone) in order to self-harm and had been admitted to hospital was afraid his zopiclone could be stopped by his GP but demonstrated a recognition of his right to be prescribed it:

And the sleeper [Zopiclone], they (hospital doctors) won’t prescribe it to me, so now I have to go back to my GP and hope that he’ll prescribe me it again, because obvious he’s gonna know on computer that I took an overdose, so he might refuse me treatment so I’ve put meself in a predicament there to try and get me medication back but I know he can’t refuse it because he’s putting me physically in danger then (ID 27, male, alcohol dependency).

### Assessment and review

Two subthemes were described under this category: medication review and discussion with clinicians.

#### Medication review

This theme came out when service users were asked about the assessment and review of their medicines with most service users describing regular review of their medicines:

Yeah, I have reviews every, I think it’s every couple of months or every few months, to see if I’m still ok on the medication, so the GP knows that it’s working basically. I get reviews ever so often. So they can know that it’s doing what it’s supposed to be doing [ID 61, female, alcohol dependency].

It [Paroxetine] has been reviewed several times and, he said “I don’t want you to come off it just yet.” and it’s due to be reviewed, so he’s [GP] going to see me and review it [ID 15, male, alcohol dependency].

By contrast, there were service users who described lack of optimal practices regarding review of their medicines:

But what happens with GPs and practices, you just become a repeat prescription, and I can go for a year without a review when they’re supposed to be every two months or every three, you know, you can be left to sort of float around. And I can ring the chemist up and say “I need another script”. And he’ll go “right, OK” and it’s there two days later. You don’t have to go see a GP. But that’s always been the case, which is not really about monitoring the effect of the drug, it’s more a case of, well you’re on it now, so just keep taking it [ID 23, male, alcohol dependency].

#### Discussion with clinicians

Service users described discussion with clinicians as important in assessing if their medicines are ‘right’ for them. They described the value of talking and listening to them:

Talking to me, listening to me, asking me, you know, why I was feeling the way I was, was there anything that had triggered things off, that sort of thing [ID 35, female, alcohol dependency].

Another service user with a particularly strong sense of entitlement to his medication (zopiclone) described how this process provided an opportunity for him to express his views on what he wants:

Oh sitting down, talking to him about sleeping tablets and what I want, it’s up to me really [ID 57, male, alcohol dependency].

On the other hand, there were service users who described not being listened to, and one service user reported the short interval in which he was prescribed a medication:

… I could do with something better for my anxiety but I don’t know what other medication I can get. I’m just taking what the doctor prescribed me. I feel like I could, it would be nice if they could give me something to help me relax more [mentions Temazepam later], you know what I mean, to calm me down but the doctor won’t prescribe anything for me like that [ID 5, male, heroin dependency].

… So I was just telling the doctor basically what my problems were and was prescribed Paroxetine, yeah, Paroxetine, I don’t think my GP probably listened and, yeah, listened and prescribed me a drug. I just went for a five minute chat with my doctor … it was just I went for a quick meeting with my GP and told him my problems and sent away with a prescription [Paroxetine]. It was just, go to the doctor and off you go, there’s your prescription off you go [ID 7, male, alcohol dependency].

## Discussion

This qualitative study aimed to explore service users’ views on the appropriateness of psychotropic medications prescribed for their co-existing mental health problems. Generally, service users described benefits from their medications (including those prescribed outside guideline recommendations) with trade-offs between their benefits and adverse effects. Service users had a sense of entitlement to being prescribed medications and seek to direct treatment decisions in line with their self-perceived needs. There were differing practices regarding the assessment and review of their medications.

Most service users described benefits from their medications, including service users who had been prescribed medications outside guideline recommendations. It was unclear whether all those prescribed outside guideline recommendations were aware of it because the interviews did not explore this issue due to its sensitive nature. Prescribing outside guideline recommendations (off-label prescribing) is common in psychiatry [34] due to the limited number of clinical trials on patients with mental health problems (especially those with complex needs) [35] as well as the non-availability of licensed medications for some psychiatric diagnoses [36]. Off-label prescribing may therefore be necessary where there is a lack of an equally safe and effective licensed alternative, informed consent from the patient to be treated off-label, sufficient evidence in the literature to support the use of the drug as well as sufficient experience of using the medicine by the prescriber [36].

Best practice recommendations including dosing and duration recommendations are usually established in clinical trials especially RCTs and where available, they serve as the foundation for clinical guidelines [37, 38] including the BNF. Where there is no evidence from clinical trials, recommendations are based to a large extent on the opinions of experts who have substantial experience in that area [39]. It may not be possible to directly extrapolate recommendations from guidelines on prescribing of psychotropic medications to people with substance misuse problems because most of the evidence from RCTs exclude them [40, 41]. Changes in the neurochemistry of the reward pathways of the brain secondary to chronic use of substances may also affect prescribing decisions [42] including dosing. Furthermore, consideration of the repercussions of relapse to substance use on the individual and society may well justify the need to sometimes prescribe outside guideline recommendations. Evidence from this study suggests that the appropriateness of prescribing is open to interpretation as some service users tended to view medications that improved their functioning as appropriate for them even when they were inappropriate by guideline standards.

While off-label prescribing may be clinically beneficial [34] for people with substance misuse problems, it also carries clinical risks such as adverse effects. For instance, prescribing supra-BNF doses of antipsychotics carries a greater risk of unwanted side effects such as extrapyramidal side effects, sedation, tachycardia, weight gain, postural hypotension, and hyperprolactinaemia [43]. The need for high doses of psychotropic medications such as anxiolytics/hypnotics may also expose the patient to the risk of dependence. In addition, people with substance misuse problems may exceed the recommended doses of these medications with the potential for overdosing [44]. Although prescribers to achieve clinical benefit with minimal risk of harm, there is sometimes conflict between balancing effectiveness and risk [45].

Service user involvement in their own care now represents a core value in the medical profession and UK National Health Service (NHS) [24, 46]. The NHS constitution states that NHS services must reflect the needs and preferences of service users [22]. However, there is sometimes conflict between physician-assessed need and what service users want, and how far prescribing should be determined by either of this remains an unresolved question [2, 45]. Service users often have complex needs and risk issues that need to be taken into consideration in treatment decisions. Service users’ choices are challenging when people have substance misuse problems because they may be less than open about the reasons why they want medications. Given that service users may be at risk from medications [13, 44, 47], prescribing decisions should be justified, and also be with due precautions that may involve limit setting, monitoring and behavioural rules in this population [44]. Nevertheless, prescribers may be under considerably greater pressure to respond to the self-perceived needs of substance users compared with other patient groups. People with substance misuse problems appear to have a particularly strong sense of their own needs and seek to make their own choices about their prescriptions. This sense of empowerment may well influence prescribing decisions.

Furthermore, there should be effective communication between the prescriber and service user for the best decision to be arrived at [48, 49]. The responses of service users in this study highlighted the fact that many of them valued this two-way communication. However, there were service users who described ineffective communication or dissatisfaction with their medications. This finding supports the results of a previous study [27] where service users with co-existing severe mental health symptoms and alcohol use disorders valued being involved in treatment decisions and expressed the need for control over them. Service users in this previous study were often dissatisfied when decisions were made without their input. Service users usually report better satisfaction, medication adherence, improved well-being, increased personal control and reduction in drug use when clinicians partner with them in decision-making [25, 26]. Health care strategies and UK guidance have also highlighted the importance of partnering with service users [23, 24].

It has been suggested that prescribing may be used as a means of terminating difficult consultations [50]. This may be more common in those with substance misuse problems, especially in time-pressured settings such as general practice [51]. This suggests that where service users with substance misuse problems do have their health conditions reviewed, GPs may prescribe without careful assessment and prescriptions may therefore be inappropriate.

There is a growing trend to use mood-altering drugs including antidepressants to treat instances of human distress or emotional unhappiness [52, 53]. It is possible that prescribing in response to life’s challenges and problems may be more common among people with substance misuse problems because of their high levels of vulnerability. Their situations are often complex with family disruptions, social and economic deprivation being prominent features [5, 54]. The lifestyle of service users usually contributes to these problems and may result in demoralisation, a sense of distress and hopelessness [44] which prescribers may be tempted to ‘fix’ with a pill. It is noteworthy that although medications are often very accessible they may not be the optimal solution. There is therefore the need for practitioners who are competent in delivering appropriate psychosocial therapies as protracted prescribing should be secondary to interventions geared to motivating people to make significant lifestyle changes. Integrated treatment by multidisciplinary teams with a focus on both the mental health disorder and substance misuse problem using a combination of pharmacotherapy and psychotherapy may also be needed. This approach has been shown to be more effective for the management of this comorbidity [55, 56].

Repeat dispensing services, where repeat supply of medications are managed by the service user’s pharmacy of choice, was introduced in the NHS community pharmacy contractual framework in 2005 in order to manage repeat prescriptions more efficiently [57]. Pharmacies are required to have appropriate governance in place for this service [58]. Service users who are suitable for repeat dispensing include those with stable therapy, long-term conditions, multiple therapies and those who can self-manage seasonal conditions [57]. This list is not exhaustive as other patient categories could be added based on clinical assessment. Repeat dispensing requires consent from the service user for their information to be shared between the dispensing and prescribing site. Recent evidence has shown that service users and prescribers value repeat prescribing and dispensing as it has the advantage of saving time, is convenient and also flexible [59, 60]. Repeat prescribing further reduces the workload of prescribers. However, there have also been concerns around repeat dispensing in primary care with majority of respondents in the report by Petty [59] stating that pharmacists never checked the items that were needed when they were ordered on behalf of the service users. The views expressed by some service users in this study clearly suggest that repeat prescribing and dispensing were not being regularly reviewed. Without on regular review of repeat medications, service users may end up being stuck on unnecessary medications and may become dependent medications that have such tendencies. Furthermore, it is also impossible to evaluate whether service users are still taking their medications with repeat prescribing. Consequently, lack of regular review of repeat prescriptions can lead to failure to detect and resolve drug-therapy problems as well as drug wastage [61].

Over the course of this study and her doctorate programme, A.O’s engagement with service users, clinicians and the literature has broadened her views on substance misuse problems. She now considers substance misuse problems to be complex disorders involving social, psychological and behavioural mechanisms.

### Strengths and limitations

To the knowledge of the authors, this is the first study to explore the views of service users attending a specialist addiction service on the appropriateness of psychotropic medications prescribed for their co-occurring mental health problems. Given that service users with substance misuse problems are difficult to recruit into research studies, a convenience sample of service users were interviewed. Consequently, the views of females and those with referral substances other than alcohol were under-represented in the interviews. Another limitation of this study is the fact that diagnoses and psychotropic medications were self-reported. There was no independent assessment of service users’ diagnoses. Most service users who took part in this study were referred from sources other than GPs to the addiction service and GP/referral notes were not available. Consequently, self-report was used as the source of information about diagnoses and prescribed medications. The findings may lack transferability to service users in other addiction services, especially given the changes that have occurred in substance misuse services in the UK. There has been an increase in the number of non-statutory and private sector providers of drug and alcohol services. Future research should involve multiple sites (including services run by the NHS and third sector organisations), to establish if the findings of this study are applicable. Furthermore, a single researcher collected study data and the researcher’s own perspectives may have affected interpretations that were made. However, the conduct, analysis and interpretation of data were overseen by two of the authors.

## Conclusion

Service users’ views concerning the appropriateness of their prescribed psychotropic medications were varied with most service users describing improved functioning as a result of their medications (including off-label prescribing). It is clear that prescribing practices around substance misuse problems and comorbidities frequently deviates from current guidelines and it is unlikely that any guideline committee would sanction the sort of pharmacotherapy. This study suggests that some prescriptions are inappropriate in terms of their usual indications but if they can be used to maintain equilibrium in service users’ lives while moving them on to recovery then such prescribing might be justified. Finally, this study points to the need for ready availability of practitioners who are competent in delivering appropriate psychosocial therapies to motivate people to making significant lifestyle changes especially in the face of protracted prescribing.
